# Interpreting systematic reviews: are we ready to make our own conclusions? A cross-sectional study

**DOI:** 10.1186/1741-7015-9-30

**Published:** 2011-03-30

**Authors:** Nai Ming Lai, Cheong Lieng Teng, Ming Lee Lee

**Affiliations:** 1 Department of Paediatrics, Monash University Sunway Campus, Jeffrey Cheah School of Medicine and Health Sciences, JKR 1235, Bukit Azah, 80100, Johor Bahru, Johor, Malaysia; 2 Department of Family Medicine, International Medical University, Jalan Rasah, 70000, Seremban, Negeri Sembilan, Malaysia; 3 Clinical Research Centre, Hospital Tuanku Jaafar, Jalan Rasah, 70000, Seremban, Negeri Sembilan, Malaysia

## Abstract

**Background:**

Independent evaluation of clinical evidence is advocated in evidence-based medicine (EBM). However, authors' conclusions are often appealing for readers who look for quick messages. We assessed how well a group of Malaysian hospital practitioners and medical students derived their own conclusions from systematic reviews (SRs) and to what extent these were influenced by their prior beliefs and the direction of the study results.

**Methods:**

We conducted two cross-sectional studies: one with hospital practitioners (*n *= 150) attending an EBM course in June 2008 in a tertiary hospital and one with final-year medical students (*n *= 35) in November 2008. We showed our participants four Cochrane SR abstracts without the authors' conclusions. For each article, the participants chose a conclusion from among six options comprising different combinations of the direction of effect and the strength of the evidence. We predetermined the single option that best reflected the actual authors' conclusions and labelled this as our best conclusion. We compared the participants' choices with our predetermined best conclusions. Two chosen reviews demonstrated that the intervention was beneficial ("positive"), and two others did not ("negative"). We also asked the participants their prior beliefs about the intervention.

**Results:**

Overall, 60.3% correctly identified the direction of effect, and 30.1% chose the best conclusions, having identified both the direction of effect and the strength of evidence. More students (48.2%) than practitioners (22.2%) chose the best conclusions (*P *< 0.001). Fewer than one-half (47%) correctly identified the direction of effect against their prior beliefs. "Positive" SRs were more likely than "negative" SRs to change the participants' beliefs about the effect of the intervention (relative risk (RR) 1.8, 95% confidence interval 1.3 to 2.6) and "convert" those who were previously unsure by making them choose the appropriate direction of effect (RR 1.9, 95% confidence interval 1.3 to 2.8).

**Conclusions:**

The majority of our participants could not generate appropriate conclusions from SRs independently. Judicious direction from the authors' conclusions still appears crucial to guiding our health care practitioners in identifying appropriate messages from research. Authors, editors and reviewers should ensure that the conclusions of a paper accurately reflect the results. Similar studies should be conducted in other settings where awareness and application of EBM are different.

Please see Commentary: http://www.biomedcentral.com/1741-7015/9/31/.

## Background

A chief desirable attribute of evidence-based medicine (EBM) practitioners is their ability to incorporate suitable clinical evidence independently into their clinical decision-making [1,2]. They understand how evidence is presented and make their own conclusions without being led by the authors. This attribute is essential to avoid following recommendations by authors who make claims that are either unsupported by their findings or are out of context [3,4]. However, some skills in EBM are usually needed in interpreting the study data, and authors' conclusions are often the most readable part of a paper. Hence, there is a possibility that clinicians with little EBM background may be directed primarily by what is written in the conclusions of a paper and carry the messages to their practise.

Other than the way the conclusions are written by the authors, the direction of the results itself may also influence the uptake of clinical evidence [5]. It has been recognised that positive studies (that is, studies that show a beneficial effect of the intervention) are more likely to be published [6,7], cited [8,9] and publicised by the media [10]. Publication bias, which follows from the selective publication of manuscripts on the basis of the strength or direction of the research findings, has been well-recognised as a major factor that gives rise to misleading results in a systematic review [11,12]. However, as far as we are aware, there has not yet been any study that assesses whether positive studies are more or less likely than negative studies to influence readers' interpretation of the results by changing their prior beliefs.

Systematic reviews (SRs) are widely accepted as the most reliable source of clinical evidence, especially regarding problems concerning a therapy [13]. They synthesise the current clinical evidence on a topic and present a single source of reference for health care decision makers, consumers and researchers [14,15]. They are cited more often than other types of studies [16]. The Cochrane Library houses a dedicated collection of SRs developed using rigorous standardised methods [17]. The quality of Cochrane SRs has been attested in comparison with SRs from other sources [18,19].

In Malaysia, there have been some coordinated efforts to promote EBM in recent years. The activities include regular nationwide workshops on EBM and Cochrane SRs, as well as exchanges between universities, to develop or strengthen their EBM curricula [20]. Despite the enthusiasm of trainers involved in the activities, it is not clear how our current and future hospital practitioners see this relatively new approach in medicine or how ready they are to practise EBM by independently interpreting the clinical evidence.

We conducted a study to assess how well a group of Malaysian hospital practitioners and medical students derived their own conclusions from the results of selected SRs. We set forth two major research questions:

1. Given only the results of a set of Cochrane SRs, what proportions of hospital staff and medical students would be able to generate the appropriate conclusions?

2. Are positive SRs, that is, SRs that show a beneficial effect of the intervention, more effective than negative SRs, that is, SRs that do not show a beneficial effect of the intervention, in changing the readers' beliefs and directing them to the appropriate conclusions?

## Materials and methods

### Study design

This was a cross-sectional study.

### Participants and settings

We recruited two groups of participants as our convenience samples. The first group, recruited in June 2008 during an introductory course on EBM, comprised hospital practitioners from Hospital Tuanku Jaafar, a tertiary hospital in Seremban, Malaysia. The hospital practitioners included clinicians of various disciplines and seniorities, as well as allied health staff (AHS), including nurses, physiotherapists, pharmacists, nutritionists and laboratory technicians. The second group, recruited in November 2008, comprised final-year medical students from the International Medical University, Malaysia. This group of students formed part of the participants in another study evaluating their interpretation of Cochrane SRs with and without authors' conclusions. The students received a structured and clinically integrated EBM training program in their final-year curriculum.

### Cochrane SR abstracts

We selected four Cochrane SR abstracts as the reading materials. The abstracts were selected on the basis of their differing direction of summary effects (benefit demonstrated (positive) or not demonstrated (negative)), the strength of the evidence (sufficient or limited), the clarity of the results, the spread of disciplines and the low likelihood of familiarity to the participants. The SR abstracts were chosen from among an initial list of 30 SRs identified in the Cochrane Library Issue 2, 2008, by the first author (NML), who read the abstracts of all the SRs and selected the first 30 that were considered potentially suitable. Two authors (NML and CLT) then short-listed nine SRs, and all three authors (NML, CLT and MLL) selected the final list of four SRs through voting and discussion. Of the four selected articles, two belonged to the Obstetrics and Gynaecology discipline, one was from the field of Neonatology and one was from Intensive Care. The SRs are listed below:

1. Alfaleh K, Bassler D: **Probiotics for prevention of necrotizing enterocolitis in preterm infants**. *Cochrane Database Syst Rev *2008, **1**:CD005496. doi:0.1002/14651858.CD005496.pub2 [21].

2. Churchill D, Beevers GD, Meher S, Rhodes C: **Diuretics for preventing pre-eclampsia**. *Cochrane Database Syst Rev *2007, **1**:CD004451. doi:10.1002/14651858.CD004451.pub2 [22].

3. Beckmann MM, Garrett AJ: **Antenatal perineal massage for reducing perineal trauma**. *Cochrane Database Syst Rev *2006, **1**:CD005123. doi:10.1002/14651858.CD005123.pub2 [23].

4. Annane D, Bellissant E, Bollaert PE, Briegel J, Keh D, Kupfer Y: **Corticosteroids for treating severe sepsis and septic shock**. *Cochrane Database Syst Rev *2004, **1**:CD002243. doi:10.1002/14651858.CD002243.pub2 [24].

For this study, we trimmed the SR abstracts to only the background, objectives and main results and deleted the authors' conclusions and other relatively standardised and technical parts, such as the search strategies, selection criteria, data collection and analysis. To achieve this, we copied and pasted each of the SR abstracts from the document in the Cochrane Library website [25] onto an empty word-processing document and deleted the unwanted parts. We used these trimmed abstracts as our materials. For each article, we highlighted one treatment-outcome combination and included the corresponding forest plot with the abstract. We asked the participants to draw their conclusions only on the highlighted treatment-outcome combination by choosing one of the following six options consisting of different combinations of direction of effect and strength of evidence:

A. It is clearly beneficial and should be recommended as a possible treatment.

B. It is clearly non-beneficial and should not be recommended as a possible treatment.

C. It appears to be beneficial from limited evidence, more studies are needed to confirm the findings.

D. It appears to be non-beneficial from limited evidence, more studies are needed to confirm the findings.

E. There is insufficient evidence to comment on whether this intervention is beneficial or not.

F. I do not understand the results presented.

We asked the participants to consider the response options based on the evidence alone, assuming that all other factors that might influence the decision to employ the intervention in practice were favourable. We also asked the participants to avoid guessing and mark option F, "I do not understand the results presented," if that was indeed the case.

Two authors (LNM and TCL) who are experienced Cochrane review authors and EBM teachers independently determined the most appropriate conclusion for each SR, using the actual review authors' conclusions as the reference standards. This was performed during our short-listing process, when we selected the final four SRs from a short list of nine SRs. In determining the most appropriate conclusion for each SR, we used the review authors' conclusions as reference standards rather than constructing our own conclusions, as we considered the authors' conclusions sufficiently robust, having gone through several rounds of peer and editorial reviews before being published. We defined "appropriate" direction of effects as the direction of effects (that is, whether the treatment benefit was demonstrated or not) that were in line with the actual finding of the SR and "inappropriate" direction of effects as one which went against the actual finding of the SR. We defined "appropriate" and "inappropriate" strength of evidence similarly, taking into consideration the number of studies or participants included, the effect size and the precision of the estimates [26]. We labelled a SR as having sufficient evidence ("clearly beneficial" or "clearly non-beneficial") if it fulfilled all of the following criteria: (1) inclusion of a sufficient number of studies or participants; (2) having a large enough effect size; (3) having precise enough estimates, judging from the width of the confidence intervals, so that the most conservative estimates at either end of the interval (depending on the direction of effect) would not appreciably change our impressions of the magnitude of the effect; (4) the review authors stated so in their conclusions. The first three criteria involved the use of subjective judgements from the raters. We defined a SR as having limited evidence if it fulfilled only some of the first three criteria and if the review authors stated so in their conclusions. We defined a SR as having "insufficient evidence to comment on whether the intervention is beneficial or not" if it did not contain any study that was eligible for inclusion and if the review authors stated so in their conclusions.

We defined the most appropriate conclusions as the conclusions with both the appropriate direction of the effects and the strength of the evidence. There was complete agreement between both authors on the most appropriate conclusions for all four chosen SRs, as follows: SR1, option A; SR2, option D; SR3, option A; and SR4, option B. Accordingly, the appropriate direction of effects for each review was determined as follows: SR1 and SR3, benefit demonstrated (option A or C); SR2 and SR4, benefit not demonstrated (option B or D). We labelled SR1 and SR3 as positive SRs because beneficial effects were demonstrated regarding the intervention examined, and SR2 and SR4 as negative SRs because beneficial effects were not demonstrated regarding the intervention examined.

### Answer sheet

The participants provided their responses on an answer sheet which was developed specifically for this study. All three authors (NML, CLT and MLL) jointly developed the items on the answer sheet after NML wrote the first draft. Apart from the predefined response options for each abstract, the answer sheet also contained the following items:

1. Discipline/department

2. The following three questionnaire items for each SR:

i. "Have you seen or heard of this review before" (response options: Yes or No)

ii. "Will the review authors' conclusions help you further in making your own conclusion on this intervention?" (response options: Yes or No)

iii. What is your belief on the intervention (for this particular outcome) prior to reading this review? (response options: "I believe that this intervention is beneficial", or "I do not believe that this intervention is beneficial", or "I am not sure")

For item 2(iii), we compared the participants' prior beliefs with the appropriate direction of effects that we determined for each review. We classified the responses into three categories: (1) Appropriate belief: belief in line with the direction of the actual SR results; (2) Inappropriate belief: belief against the direction of the actual SR results; and (3) Unsure. We did not incorporate the strength of the evidence into our question for this item to avoid possible confusion among the readers as a result of having to deal with two elements (direction of effects and strength of evidence) in their recall when answering this question. The terms "appropriate" and "inappropriate" used throughout the paper are working terms assigned for this study only.

### Conduct of the study

We briefed all participants on the voluntary and anonymous nature of the study. We took the completion of anonymous answer scripts as consent to participate in the study. We asked any participant who would prefer not to participate in the study to return a blank answer sheet. We informed the medical students that their decision to participate would not influence their university standing. For the hospital practitioners, we conducted the study midway into the introductory EBM course, and for the medical students, we conducted the study midway into the final six months of their undergraduate medical training. All participants received instructions on interpreting Cochrane SR abstracts prior to the study. For hospital practitioners, we conducted a brief lecture during the introductory EBM course on understanding common EBM expressions such as RR, 95% confidence interval, interpreting a forest plot and determining the direction of effects and the strength of evidence using an unrelated Cochrane SR abstract as an example. The medical students had received relevant instructions in their EBM training program which was incorporated into their final six months of medical training. The instructions covered the interpretation of Cochrane SRs, including how to determine the direction of effect and the strength of evidence.

During the study, we projected the four selected SR abstracts sequentially on a screen for the hospital practitioners to view, with approximately 5 minutes allocated for each SR. The medical students received printed copies of the trimmed SRs to read. Prior to the study, we instructed the participants not to communicate with each other, and an administrative staff member monitored them throughout the study.

### Ethics approval

The study was approved by the Clinical Research Centre, Hospital Tuanku Jaafar, Seremban, Malaysia, and the Research and Ethics Committee, International Medical University, Malaysia. The study was also registered with the National Medical Research Registry, Malaysia.

### Statistical analyses

We performed cross-tabulations using χ^2 ^tests to compare differences in responses in addition to descriptive statistics using SPSS version 15 (SPSS Inc., Chicago, IL, USA).

## Results

A total of 185 participants were approached, including 150 hospital practitioners and 35 medical students. Overall, 130 participants (70.3%) consented to take part, including 95 hospital practitioners (44 clinicians and 51 AHS), for a 63.3% response rate) and 35 medical students (100% response rate). Each participant read through four Cochrane SR abstracts, adding up to a total of 520 encounters.

### Assessing the ability to identify the most appropriate conclusions

**Figure 1:**
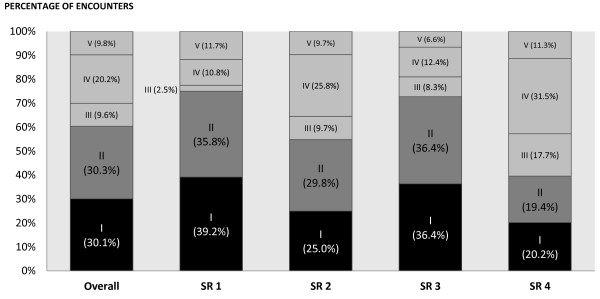
**Directions of effects and strength of evidence in the participants' postreading conclusions**. **(a) **Proportion of participants with the appropriate direction of effects and strength of evidence (that is, the most appropriate conclusions) (shaded in black). **(b) **Proportion of participants with the appropriate direction of effects, but inappropriate strength of evidence (shaded in dark grey). **(c) **Proportion of participants with the appropriate strength of evidence, but inappropriate direction of effects (shaded in grey). **(d) **Proportions with both the inappropriate direction of effects and strength of evidence (shaded in grey). **(e) **Proportion of participants who did not understand the results (shaded in grey).

Figure 1 shows the responses of the participants categorised according to the appropriateness of their postreading conclusions in terms of the direction of effects and the strength of evidence. The darker shaded bars in the figure illustrate the proportions of participants who identified the appropriate directions of effects. Overall, in 60.4% of the encounters, the participants identified the appropriate direction of effects (30.1% as represented by the black bar and 30.3% as represented by the dark grey bar). Looking at the four SRs separately, higher proportions identified the appropriate directions of effects for SR1 and SR3, that is, the positive SRs (75.0% and 72.7%, respectively) compared to SR2 and SR4, that is, the negative SRs (54.8% and 39.5%, respectively) (Figure 1).

We considered the conclusions with both the appropriate direction of effects and strength of the evidence as the most appropriate conclusions. As shown in Figure 1, in 30.1% of the encounters, the participants identified the most appropriate conclusions (as represented by the black bar). Looking at the four SRs separately, higher proportions of participants identified the most appropriate conclusions in SR1 and SR3, that is, the positive SRs (39.2% and 36.4%, respectively), compared to SR2 and SR4, that is, the negative SRs (25.0% and 20.2%, respectively) (Figure 1). More medical students (48.2%) than hospital practitioners (22.2%), clinicians (21.7%) and AHS (22.6%) identified the most appropriate conclusions (*P *< 0.001).

In 20.9% of the encounters, the participants indicated that they had seen or heard of the SRs before. However, these participants were not more likely to identify the most appropriate conclusions than those who reported having no prior impression of the SRs (16.3% versus 22.9%; relative risk (RR) 0.74, 95% confidence interval (95% CI) 0.52 to 1.08).

### Assessing the influence of prior beliefs on the post-reading conclusions

Table 1 shows the postreading conclusions of the participants compared with their prior beliefs. In 203 encounters (41.5%), the participants' prior beliefs were in line with the appropriate directions of effects. In 149 encounters (30.5%), the participants' prior beliefs went against the appropriate direction of effects, and in the remaining 28.0% of the encounters the participants were unsure about the effect of the intervention prior to reading the SRs. After reading the SRs, fewer than one-half (47.0%) of participants who had the inappropriate belief changed their beliefs and chose the appropriate direction of effects. On the other hand, 19.2% of those who had the appropriate prior beliefs appeared to have been misled by the SRs and chose the inappropriate conclusions (Table 1).

**Table 1:** Postreading conclusions evaluated in comparison with prior beliefs of the participants^a^

	Prior beliefs			
	
Postreading conclusions, *n *(%)	Appropriate	Inappropriate	Unsure	Total
Appropriate	163 (80.3%)	70 (47.0%)	62 (45.3%)	295 (60.4%)
Inappropriate	39 (19.2%)	74 (49.7%)	33 (24.1%)	146 (29.8%)
Did not understand	1 (0.5%)	5 (3.3%)	42 (30.7%)	48 (9.8%)
Total	203 (100%)	149 (100%)	137 (100%)	489 (100%)

^a^All numbers and percentages are based on the number of encounters. Percentages shown are for each column. "Appropriate" means in line with the direction of the reference standards, and "inappropriate" means the opposite of the direction of the reference standards. The overall level of agreement between postreading conclusions and prior beliefs were κ = 0.22 ± 0.03 (SE) and *P *< 0.001.

### Influence of positive and negative SRs on the participants' postreading conclusions

**Figure 2:**
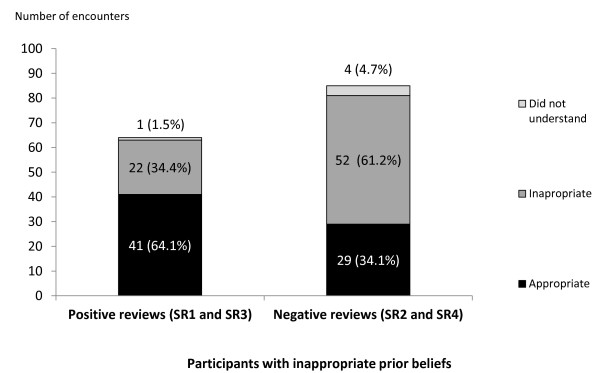
**Postreading conclusions of participants with inappropriate prior beliefs**. The responses after reading the positive systematic reviews (SRs) (SR 1 and SR 3) are shown in the stacked bars on the left, and the responses after reading the negative SRs (SR 2 and SR 4) are shown in the stacked bars on the right.

We assessed the influence of the positive and negative SRs in changing the beliefs of two groups of participants: (1) participants who had inappropriate prior beliefs regarding the effects of the interventions and (2) participants who were unsure of the effects of the intervention prior to reading the SRs. Figure 2 shows the responses of these two groups of participants after reading the positive and negative SRs.

**Figure 3:**
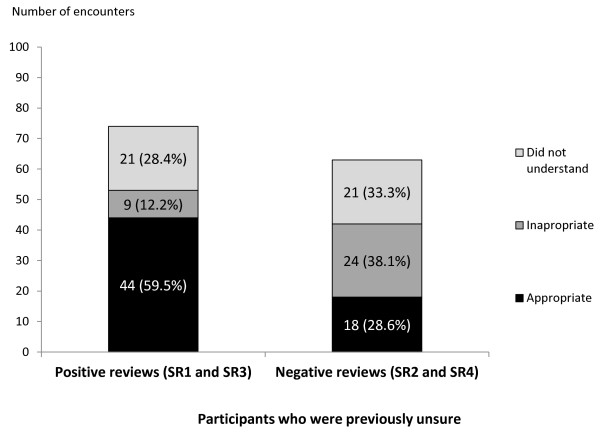
**Postreading conclusions of participants who were previously unsure**. The participants' responses after reading the positive SRs (SR 1 and SR 3) are shown in the stacked bars on the left, and the participants' responses after reading the negative SRs (SR 2 and SR 4) are shown in the stacked bars on the right.

After reading the positive SRs (SR1 and SR3), nearly two-thirds (64.1%) of participants who had held inappropriate prior beliefs (that is, believing that the intervention was nonbeneficial) (Figure 2) and 59.5% who were previously unsure (Figure 3) changed their beliefs and concluded appropriately that the interventions were beneficial. In contrast, after reading the negative SRs (SR2 and SR4), only about one-third (34.1%) of the readers who had held inappropriate prior beliefs (that is, believing that the intervention was beneficial) (Figure 2) and 28.6% who were previously unsure (Figure 3) changed their beliefs and concluded appropriately that the interventions were not beneficial.

On the basis of these findings, we concluded that, overall, our participants who had inappropriate prior beliefs were more likely to change their beliefs and choose the appropriate conclusions after reading the positive SRs compared to the negative SRs (RR 1.8, 95% CI 1.3 to 2.6). Participants who had previously been unsure were also more likely to choose the appropriate conclusions after reading the positive SRs compared to the negative SRs (RR 1.9, 95% CI 1.3 to 2.8).

Among the 19.2% who had appropriate prior beliefs but appeared to have been misled by the reviews by choosing the inappropriate conclusions (Table 1), significantly more did so after reading the negative SRs than the positive SRs (29% versus 9.7%, *P *= 0.001).

### Perceptions of the value of the authors' conclusions

Two-thirds (68.7%) of the participants indicated that the authors' conclusions, had they been available, would have helped them in drawing their own conclusions. Interestingly, more of them identified the most appropriate conclusions compared to those who considered the authors' conclusions unnecessary, although the difference did not reach statistical significance (32.2% versus 27.2%, *P *= 0.3).

## Discussion

Our results suggest that the majority of our participants could not interpret research findings accurately in the absence of guidance provided by the authors' conclusions, and positive SRs appeared to have greater influence than negative SRs in directing the readers' postreading conclusions.

A previous study showed that when presented with identical primary data, the conclusions among researchers varied significantly [27]. Another study that examined nearly 300 Cochrane and non-Cochrane SRs found that in a significant proportion of the SRs, the review authors drew conclusions that were not entirely in line with their results as assessed by the third party [28]. The findings of these two studies [27,28] highlight the challenges that researchers face in interpreting even their own work, as well as the importance of being independent in interpreting the results of a study and generating conclusions that are justifiable and appropriate to one's clinical circumstances. In our study, the participants were not researchers, but a group of end users of the literature, including practitioners from a Malaysian tertiary hospital and senior medical students from one of the largest medical schools in Malaysia. We provided synthesised, readily interpretable data in the form of published abstracts, which were what they were likely to read in practice. However, after reading the SRs, nearly one-half of our participants failed to identify the appropriate direction of effect, and less than one-third accurately judged both the direction of effects as well as the strength of evidence. This suggests a lack of basic skills in interpreting research evidence. If such problems exist in interpreting Cochrane SRs, which are written in a relatively standardised manner, other forms of articles may pose even greater difficulties in view of their varied presentations. In this study, medical students fared better than hospital practitioners, possibly as a result of their exposure to EBM in the curriculum, as EBM has been a major component in the undergraduate curricula of the Malaysian medical schools only in the past decade. The full participation rate from the medical students compared to the relatively poor participation rate from the hospital practitioners might be a reflection of the difference in their interest in EBM, which might also have contributed to the differences in their performances. Our findings also somewhat echo those of earlier studies which showed that undergraduate training in EBM appeared to produce greater gains in learning compared to EBM training for practising clinicians [29].

In our study, positive SRs appeared to have greater impact than negative SRs in directing readers' conclusions. Readers who held inappropriate prior beliefs appeared to "convert" more easily after reading positive SRs compared to negative SRs, and those who were previously unsure seemed more likely to commit to the appropriate conclusions after reading the positive SRs. Not only were the negative SRs less convincing, but also, they seemed more likely to mislead, as more readers who had the appropriate prior beliefs (believing that the intervention was nonbeneficial) chose the inappropriate conclusions (indicating that the intervention was beneficial) after reading the negative SRs compared to the positive SRs. It was likely that our participants' lack of EBM knowledge made them prone to extraneous influences from the studies. Our findings also add to the existing literature on the differential impacts of positive versus negative studies [6].

Another interesting finding is that more participants who considered the authors' conclusions helpful made the appropriate conclusions themselves compared to those who did not think the conclusions would help them further. The findings might well be explained by differences in the levels of interest in the subject matter, which may be minimised in future studies by choosing a homogeneous group of participants rather than a group with mixed backgrounds such as those recruited into the present study.

### Strengths and limitations

As far as we are aware, our study is the first to examine the ability of a group of end users (hospital practitioners and medical students) in interpreting research independently. We used Cochrane SRs in the hope that their relatively standardised presentation format would reduce the confounding effects caused by variations in the quality of presentation in determining participants' understanding of the results. We used four SRs as the reading material instead of a single SR, which probably increased the reliability of our principal findings. We demonstrated that participants with prior impressions of the SRs were not more likely than those without prior impressions to choose the most appropriate conclusions, thus accounting for any possible influence that prior impressions had on their interpretation of the data.

However, we note the following limitations of our study in terms of methods and applicability. First, in terms of methods, we did not formally pilot our questionnaire, so there might be some differences between what we actually measured and what we intended to measure. Second, we conducted the study slightly differently between the hospital staff and the medical students, with the former reading the SR abstracts projected onto a screen and the latter reading the SR abstracts in printed form. However, we do not think that these factors would have introduced substantial bias in terms of the difference in total reading time, as the total allocated time for reading the SRs were 20 minutes for the hospital practitioners, while all medical students completed the study within 20 minutes. Next, as we assessed the participants' prior beliefs and asked them to read the SRs at the same time, the participants' responses regarding their prior beliefs might have been influenced by their postreading impressions of the SRs. However, the biases introduced as a result would most probably have attenuated the differences between the influences of positive and negative SRs by apparently decreasing the number who chose the conclusions against their beliefs. In our study, the difference remained substantial despite the possible biases. Nevertheless, to minimise such biases, we suggest that in future studies, questionnaire items assessing prior beliefs may be asked separately, before the participants read the articles. Next, we acknowledge that the participants' responses might have incorporated their own perceptions of the clinical importance of the effect sizes, which we could not measure, despite the fact that we selected the positive SRs with either large effects (SR1) or precise estimates (SR3) and asked the participants to consider the data alone when drawing their conclusions. Additionally, the small number of SRs from a narrow range of disciplines might have limited the generalisability of our findings.

In terms of applicability, the hospital practitioners and medical students in this study were recruited from a large tertiary hospital and a large medical school, respectively, and they might well represent the relevant populations in Malaysia with regard to their exposure to EBM. However, our sample might not be representative of the hospital practitioners and medical students in other countries, where the awareness, competence and application of EBM are different. Additionally, there have been changes in the presentation format of the Cochrane SRs since the time of our study in 2008. In particular, a new feature in the Cochrane SRs, the "summary of findings" table, has recently been introduced to provide a quick reference on the effect sizes and the quality of evidence [30]. It would be interesting to run a similar study using the SRs with the "summary of findings" tables incorporated.

## Conclusion

In summary, this study has shown that a group of current and future health care practitioners in Malaysia had difficulties in generating the appropriate conclusions from SRs independently. This implies that, at least in our setting, judicious direction of authors in their conclusions still appears crucial to guiding our health care practitioners in identifying appropriate messages from research, and efforts to strengthen EBM training in general appear necessary. This in turn highlights how important it is for study authors, journal editors and peer reviewers to ensure that the conclusions of a paper are appropriate and supported by the study data. The present study serves as a primer for similar studies in other settings in which the awareness and application of EBM may be different. Further work is also needed to explore how factors such as prior beliefs and the direction of the study results influence the interpretation of clinical data.

## Competing interests

NML and CLT are Cochrane review authors. The three authors have no other financial or nonfinancial interests relevant to the paper.

## Authors' contributions

NML conceived the study, drafted the research proposal, obtained ethics approval and funding from the International Medical University, Malaysia, selected the study materials, designed the answer sheet and data collection tools, implemented the study with medical students, monitored data collection for the whole trial, wrote the statistical analysis plan, cleaned and analysed the data and drafted and revised the paper. He is the guarantor. CLT cowrote the research protocol, selected the study materials, assisted in data analysis and interpretation and revised the paper. MLL obtained ethics approval from Hospital Tuanku Jaafar, registered the study in the Malaysian National Medical Research Registry, implemented the study with hospital practitioners, assisted in data interpretation and drafted the paper. 

## Authors' information

NML is a Senior Lecturer in Paediatrics at the Monash University Sunway Campus, Johor Bahru, Johor, Malaysia. He is involved in teaching and assessing students in the Evidence Based Clinical Practice (EBCP) module in years three and four. He was formerly with the International Medical University, Seremban, Negeri Sembilan, Malaysia. CLT is a Professor of Family Practice and the main coordinator of the Evidence Based Medicine curriculum at the International Medical University, Seremban, Negeri Sembilan, Malaysia. MLL is the Head of Clinical Research Centre at the Hospital Tuanku Jaafar, Seremban, Negeri Sembilan, Malaysia.

## Pre-publication history

The pre-publication history for this paper can be accessed here:

http://www.biomedcentral.com/1741-7015/9/30/prepub
